# Effect of saffron on rat sperm chromatin integrity

**Published:** 2014-05

**Authors:** Mohammad Mardani, Ahmad Vaez, Shahnaz Razavi

**Affiliations:** *Department of Anatomical Sciences and Molecular Biology, School of Medicine, Isfahan University of Medical Sciences, Isfahan, Iran*

**Keywords:** *Antioxidants*, *Saffron*, *Vitamin E*, *DNA damage*, *Sperm chromatin*

## Abstract

**Background:** Currently, relation between reactive oxygen species (ROS) ROS concentration and semen quality was indicated. Saffron has traditionally been not only considered as a food additive but also as a medicinal herb, which has a good antioxidant properties.

**Objective:** The aim of this study was to evaluate the protection potency of saffron and vitamin E on sperm chromatin integrity.

**Materials and Methods: **Thirty adult male Wistar rats divided equally into saffron (100 mg/kg), vitamin E (100 mg/kg) and control (0.5cc distilled water /day) groups. After 60 days, cauda epididymis dissected and sperm cells were used for analysis of sperm chromatin packaging by chromomycin A3 (CMA3) staining, and sperm chromatin susceptibility to acid denaturation by acridine orange (AO) staining.

**Results: **The mean percentage of CMA3 positive sperm was significantly decreased in saffron and vitamin E groups relative to control group (p<0.001). Moreover, the AO staining results showed that the mean percentage of sperm with DNA damage was significantly decreased in saffron and vitamin E groups as compared with control group (p<0.001).

**Conclusion:** Our results purposed that saffron can protect sperm against DNA damage and chromatin anomalies.

## Introduction

About 50% of infertility cases are related to male factor(Dohle, 2005 #134). In addition, anatomical abnormalities, endocrine disturbances, infections, psychological, immunological, environmental, and genetic factors play important roles in infertility (1). One of the main factors, which has been studied extensively in recent years and can affect fertility, is oxidative stress (OS). OS is a condition with excessive production of Reactive Oxygen Species (ROS), which is associated with antioxidant system failure (2, 3). All cellular components such as nucleic acids, sugars, proteins and fatty acids can be destroyed by OS (4). 

It also has shown that excessive ROS causes DNA fragmentation, which leads to failure of fertilization (5, 6). Sperm chromatin condensation is one of the main strategies against OS harmful effects. During spermatogenesis phase, histones, usual chromatin packaging proteins, are replaced first by transition proteins and then by protamine’s, small, arginine/cysteine -rich, and highly basic proteins. As a result, sperm chromatin is highly compacted. During the transit of sperm through the epididymis, the cysteine-thiol groups of protamine’s are progressively oxidized and covalent disulphide bonds (S-S) are formed. In the cases of reduction in the number of S-S bonds between protamine molecules, the chromatin will be more susceptible to denaturation (7). 

On the other hand, deficiency of zinc can affect chromatin packaging; zinc secreted from prostate may also play an important role in contributing to the formation of non-covalent S-Zn-S bonds, which additionally stabilize the sperm chromatin structure. Therefore, sperm become highly resistant against variety of detergents after nuclear chromatin condensation (8). Indeed, various reports in mammalian species support the hypothesis of a close relationship between nuclear maturity and fertility of ejaculated sperm (9). Sperms are protected against OS by three different antioxidant protection systems: endogenous antioxidants, dietary antioxidants, and metal-binding proteins (5, 10-13). 

Endogenous antioxidants comprise enzymatic and non-enzymatic antioxidants. Enzymatic antioxidants include superoxide dismutase (SOD), catalase, and glutathione peroxidase (GPX), glutathione reductase (GRD) and non-enzymatic antioxidants contain ascorbate, vitamin E, vitamin A, glutathione, pyruvate, albumin, ubiquitol, taurine, and hypotaurine (10, 11). Dietary antioxidants (dietary supplements) are usually present in the form of vitamin C, vitamin E, beta-carotenes, carotenoids, and flavonoids (13). Metal-binding proteins such as albumin, ceruloplasmin, metallothionein, transferrin, ferritin, and myoglobin function by inactivating transition metal ions that otherwise would have catalyzed the production of free radicals (12).

One of the most important antioxidants is vitamin E (alpha-tocopherol) which is residing mainly on the cell membrane and inside the cell, interrupts with lipid peroxidation by scavenge free radicals, which is suggested as ''a major chain breaking antioxidant'' (14). Also vitamin E plays an important protective role against OS by reducing malondialdehyde level and improving the antioxidant defense system activity in testicular cells (15). Furthermore, it is believed that vitamin E is the primary components of the antioxidant system of the spermatozoa (16). It is demonstrated the combined of compound Xuanju capsule and vitamin E can increase seminal quality and decrease sperm chromatin damage in oligoasthenozoospermia men (17).

Saffron, with well-known antioxidant properties, is dried stigma of flower of saffron (Crocus sativus L.) that belongs to Iridaceae family. Saffron is cultivated in Iran, Spain, Italy, Greece and India. Because of its color, taste and smell it is used as a food additive. Saffron has traditionally been considered as a medicinal plant. Previous studies have demonstrated a wide variety of pharmacological effects of saffron and its active constituents. Some of therapeutic effects of saffron and its constituents include: antioxidant properties, sexual potential stimulant, free radical scavenger, anti-mutation trait, genetic protection effect, and sperm morphology and motility improvement (18-23).

Due to increasing implementation of medicinal plants in treatment of infertility, this study was designed to examine the influence of saffron (Crocus sativus L. stigma) as an antioxidant and vitamin E as a positive control on rat sperm chromatin packaging and DNA damage.

## Materials and methods

**Experimental animals**

This experimental study was carried out on 30 mature (12 weeks old) male Wistar rats (180-200 gr) obtained from Pasteur Institute, Tehran, Iran. All animal procedures were approved by our university ethical committee. For 10 days, animals were acclimatized before experiment. 

Rats were kept in colony cages with 21±2^o^C temperature and under12 hours light and 12 hours dark conditions and with access to laboratory pellet chow and water *ad libitum*. Rats were divided randomly and equally into three following groups: saffron, vitamin E and control groups receiving, saffron 100 mg/kg/day, vitamin E 100 mg/kg/day and distilled water 0.5 ml/day via ora-gastric catheter, respectively. After 60 days, rats first were anesthetized with chloroform and then cervical dislocation was done.

**Saffron and vitamin E**

Saffron obtained from farmland of Ghaen-Khorasan- Iran. For preparation 100 mg/kg saffron according to Khayatnouri *et al* procedure, 1000 mg grinded saffron was soaked and stirred in 20 mL warmed distilled water for at least two hours (24). The solution was stored at 4^o^C until further use. This solution was used daily for treatment of saffron group rats. Vitamin E purchased from Sigma Chemical Co, St. Louis, Mo. Previous studies showed that 100 mg vitamin E/kg body weight in rats was effective dose (25).

**Epididymal sperm preparation**

After rat dissection, left epididymis was removed immediately. Distal end of the cauda epididymis was separated and placed in petri dish contain 1 mL of pre-warmed (37^o^C) normal saline. Several incisions were made in tissue; sperm were allowed to swim out into the dish. The sperm suspension was incubated for 10 min.

**Sperm parameters**

The sperm count and motility was determined using Neubauer's chamber (LABART, Germany) and phase-contrast microscope (Nikon TE2000-U, Japan) at magnification ×200. 

One ml of epididymis suspension was added to 9 ml normal saline. An aliquot of the diluted suspension is put into the Neubauer's chamber, and sperm heads was manually counted (26). For sperm motility, data was expressed as percentage of motile and non-motile spermatozoa. Total sperm morphological abnormalities were assessed using phase-contrast microscopy. For each sample, 200 sperm were assessed for head, neck, and tail abnormalities (Table I).

**Sperm chromatin integrity**

**Chromomycin A3 (CMA3) staining**

CMA3 staining was performed based on Razavi* et al* with minor modification (27). After preparing a suitable concentration of sperm suspension, smears were first prepared, air dried; and then were fixed in Carnoy's solution (methanol/glacial acetic acid, 3:1) at 4^o^C for 10 min. Before staining each smear was incubated with acid-detergent solution pH=1.2 [0.08 NHCl, 0.15M NaCl, 0.1% Triton-X 100], then each slide was treated with 100 mL of CMA3 (0.25 mg/mL in McIlvain buffer; 7 mL citric acid 0.1+32.9 mL Na_2_HPO_4_ 7H_2_O 0.2 M, pH 7.0 containing 10 mM MgCl_2_), (Sigma, St Louis, MO, USA) for 20 min. 

**Figure 1:**
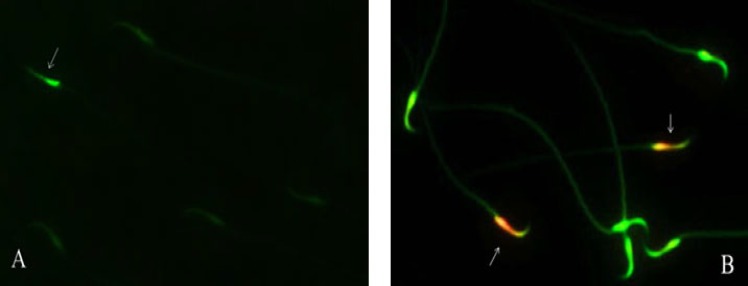
Photograph of sperm chromatin status. A: Chromomycin A3 staining; arrow shows sperm chromatin deficiency (CMA3+), B: Acridine orange staining; arrows show sperm with DNA damage (AO+).

**Figure 2:**
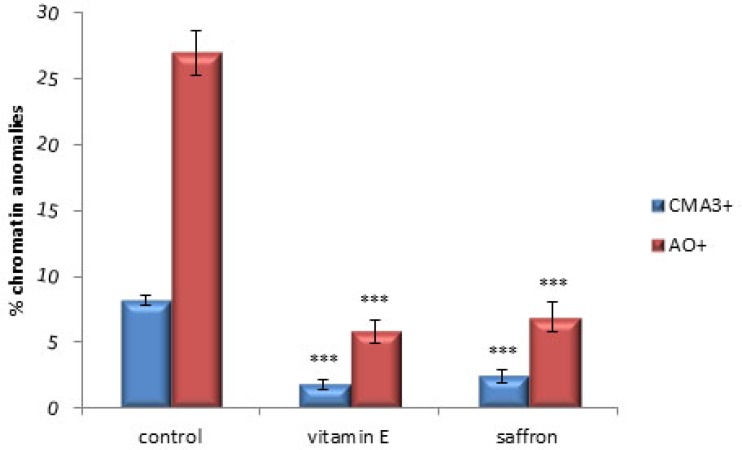
The mean percentage of Chromomycin A3 positive (CMA3+) and Acridine orange positive (AO+) sperm in different groups (n=30). ***: p<0.001. One-Way ANOVA followed by Tukey's post hoc test

At the end, slides were washed in buffer and mounted with buffered glycerol (1:1). Microscopic analysis of the slides was performed by fluorescent microscope (OLYMPUS BX51-Japan), with an appropriate filter (460 nm) (27, 28). Evaluation sperm was done by distinguishing between bright green stained as a sperm CMA3 positive and dull green or colorless stained sperm as CMA3 negative (Figure 1A, Figure 2).

**Acridine orange staining**

We carried out this protocol according to Nasr-Esfahani *et al* with minor modification (29). After procurement a suitable concentration of sperm suspension, smears were prepared, then before staining each smear was incubated with acid-detergent solution pH= 1.2 [0.08 N HCl, 0.15 M NaCl, 0.1% Triton-X 100], then was stained for 10 min with freshly prepared acridine orange (0.19 mg/mL) in citrate phosphate buffer (80 mL citric acid 0.1 M+5 mL NaH_2_PO_4_ 0.3 M, pH= 2.5). Smears were evaluated on the same day by fluorescent microscope with 460 nm filter. The duration of illumination was limited to 40 second/field. The percentage of green (normal double stranded DNA) and orange/red (abnormally denatured DNA) fluorescence sperm per sample was calculated (Figure1B, Figure 2).

**Statistical Analysis**

Data were analyzed using Statistical Package for the Social Sciences (SPSS) 17.0 software. All data were expressed as Mean±SE. Data were compared with One-Way ANOVA followed by Tukey's post hoc test for multiple comparisons. Data were considered statistically significant when p<0.05.

## Results

**Sperm parameters**

The mean percentage of sperm concentration in control group didn’t have statistically significant difference with experimental groups. The mean percentage of sperm motility was significantly increased in both experimental groups relative to control group (p<0.001); however, significant difference in the mean percentage of motility was observed between saffron and vitamin E groups (p<0.05). The mean percentages of total abnormal morphology in experimental groups was decreased compared with control group (p<0.001) (Table I).

**Chromomycin A3 (CMA3) staining**

The mean percentage of sperm with protamine deficiency (CMA3‏+) in control group was 8.16±0.34 that significantly decreased to 1.78±0.43 and 2.44±0.50 in saffron and vitamin E groups respectively (p<0.001) (Figure1A, Figure 2). 

**Acridine orange (AO) staining**

The mean percentage of DNA damaged sperm (AO^+^) in control group was 26.98±1.64 that significantly decreased to 5.82±0.85 in vitamin E group, while the mean percentage of DNA damaged sperm (AO^+^) in saffron group was 6.93±1.13 (p<0.001) (Figure 1B, Figure 2).

**Table I:** Motility, abnormality and concentration of rat sperm in control, vitamin E and saffron groups (n=30)

**Sperm parameters**	**Groups**		
	**Control**	**Vitamin E**	**Saffron**
Concentration (×10^6^)	88.73 ± 10.66	112.65 ± 15.28	97.40 ± 12.40
Motility (%)	5.90 ± 0.61	12.51 ± 1.27***	17.95 ± 1.43***,*^a^
Total abnormal morphology (%)	39.28 ± 1.45	17.38 ± 1.50***	19.96 ± 1.33***

Note: Values are expressed as means±SE. One-Way ANOVA followed by Tukey's post hoc test

*: P < 0.05,

*** : P < 0.001, compared with control group.

a : compared with vitamin E group.

## Discussion

The present study has evaluated the effect of saffron and vitamin E on rat sperm parameters and chromatin integrity. Our results revealed that saffron and vitamin E can protect sperm chromatin and decline susceptibility of DNA to damage after exposure to acid-detergent solution. Also saffron and vitamin E can ameliorate sperm motility and decrease abnormal morphology. In this study, we applied saffron and vitamin E as antioxidant; also CMA3 and AO staining, to evaluate chromatin status. CMA3 staining is used to show chromatin packaging quality of sperm; in which high CMA3 positivity is an indicator for low sperm protamination conditions (30). Our previous study showed that protamine deficiency is correlated with fertilization rate in IVF and ICSI cycles (9, 28). AO is a biomarker of the sperm nuclear DNA susceptibility to in situ acid-induced denaturation. Monomeric AO sits on the double-stranded DNA, whereas the aggregation of AO binds to single-stranded DNA (29, 31). 

In our previous study, it was established that sperm DNA damages can contribute to poor quality of subsequent embryo development (9, 32). Of many factors that can contribute to infertility, OS is recently noted. OS is a condition of balance disturbance between oxidants and cell antioxidant system, which free radicals can attack to all the cellular components (2, 4). OS can produce DNA damage in the form of modification of all bases includes: changing, shifting, free sites creation, and deletion (33). If damage is minor, sperm (also oocyte) is capable to repair it, but in extensive damage, apoptosis and embryo fragmentation can occur (4, 34). 

Antioxidants protect sperm against ROS by neutralization of free radicals. Antioxidants can protect DNA and other cellular components, which can lead to semen quality improvement (35). Recently, considerable attentions have been focused on the evaluation of dietary antioxidant and medicinal herbs for infertility treatment. Also antioxidant properties of saffron were documented in several studies before (18). 

The antioxidant activity of saffron is due to its derivatives (safranal, crocin, crocetin, dimethyl crocetin); each of them has individual antioxidant trait, however the synergistic effect of them gives saffron a significant antioxidant potential (36). Saffron has aphrodisiac properties and hence used in folk medicine to cure impotence. It was shown that saffron and its bioactive ingredients could increase libido, enhancement of erectile function, and amelioration of semen quality (23, 37). Hosseinzadeh and colleagues showed that intraperitoneally administration of saffron ameliorates sexual behaviors such as erection frequency, mounting frequency, intromission frequency and conversely reduce mount latency, intromission latency and ejaculation latency parameters (37).

Our results revealed that saffron didn’t have statistically influence on rat sperm concentration; but significantly improved motility and normal sperm morphology, these results are consistent with Heidary* et al. *They reported that applying 50 mg saffron 3 times a week for 3 months, significantly increases normal human sperm morphology and motility (p<0.001), but they didn’t observe significant difference in sperm concentration (23). In accordance with our results, Dominguez-Rebolledo and coworkers applied 1 mM or 0.1 mM of different antioxidants (lipoic acid, melatonin, Trolox and crocin) with or without OS (100 muM Fe^2+^) on red deer cryopreserved frozen thawed sperm at 37^o^C. They reported that Trolox and crocin had maximum antioxidant protection. Crocin similar to Trolox especially at 1 mM ameliorated motility and had no effect on lipid peroxidation (38). 

However, Safarinejad *et al *show that administration of 60 mg/day saffron didn’t have any positive effect on sperm parameters (density, morphology and motility) in infertile men with idiopathic oligoasthenotera tozoospermia (OAT) compared with placebo. Also saffron does not significantly improve total seminal plasma antioxidant capacity of idiopathic (OAT) patient in comparison with control group (39). Dissimilarity with our results could be due to different dose and kind of samples. In addition, the genetic protection effect of saffron has reported by several studies. Premkumar *et al* showed that pretreatment with different doses of saffron (20, 40, and 80 mg/kg) in mice can significantly decline DNA damage induced by anti-tumor drugs (22). 

Hosseinzadeh* et al* showed that using 40 and 80 mg/kg saffron and 50, 200 and 400 mg/kg crocin 45 min prior to methyl-methanesulfonate administration can protect liver, kidney, lung, and spleen against DNA damage (p<0.001) (40). On the other hand, the effect of vitamin E on reproductive health is so clear, which is considered as an ''anti-sterility vitamin'' (41). Vitamin E seems to be a major chain-breaking antioxidant, which locate within the seminal plasma and plasma membrane (14). Our results showed that vitamin E increased sperm motility and normal morphology, but didn’t have significant effect on sperm concentration. In agreement, Suleiman *et al* reported that administration of 300 mg vitamin E in patient with asthenozoospermia led to a significant increase in sperm motility (35). 

Keskes-Ammar *et al* found that orally administration 400 mg vitamin E and 225 mg selenium caused to statistically significant increase in infertile men's sperm motility and concentration (42). Also, Paradiso Galatioto and coworkers showed that antioxidant therapy with N-acetylcysteine (NAC) 600 mg and vitamins-minerals causes to significant increase on sperm concentration of patient with oligozoospermia (43). Moreover, Momeni and colleagues administered vitamin E alone and with para-nonylphenol (p-NP) in rats. They reported that vitamin E ameliorates sperm viability in vitamin E and p-NP compared to control group. In addition, sperm number, and progressively motile sperm were improved in combined p-NP with vitamin E group compared with p-NP group (44). In contrast, Kessopoulou *et al* obtained no significant changes in human sperm concentration, motility, and morphology after 3 months treated with 600 mg vitamin E (45).

Dissimilarity with our outcomes could be due to different dose administration, kind of samples and experiment period. Previous studies indicated that vitamin E as well as other antioxidants can protect DNA. In agreement with our chromatin protective results of vitamin E, Greco *et al* found that 1 gr/day vitamin C and E causes to significant decrease in sperm DNA damage (p<0.001), but they didn’t find any significant differences in sperm parameters (46). Also Tunc* et al* reported however there were no significant changes in sperm parameters (concentration, motility, and morphology) of infertile men which treated by Menevit (lycopene, vitamins, zinc, Se, folate, garlic) for a period of 3 months, but sperm chromatin integrity and protamine packaging improved significantly (47). While, Menezo *et al* reported that applying vitamins C, E (400 mg), zinc, Selenium caused to decrease in sperm DNA fragmentation index (DFI) (p<0.001) with an unexpected increase in sperm decondensation (p<0.001). 

They concluded that the negative effect on sperm decondensation may be due to antioxidants action, especially vitamin C, on cysteine disulfide bridges of protamine (48). In contrast with our results, Piomboni and coworkers reported that applying beta-glucan, papaya, lactoferrin, and vitamins C and E, significantly increases progressive motility and normal morphology, but no statistically significant changes in sperm DNA damage were repeated (49).

## Conclusion

In conclusion saffron has a positive effect on DNA damage and protamination in rat sperm. As saffron comprises various active constituents, further studies are needed to define their mechanism of action and effective dosage in human. Nevertheless, we acknowledge the need for further studies on the other sperm genetic aspects. 

## Footnote

***This article extracted from M.Sc. thesis. (Ahmad Vaez)***
